# Absolute copy number differences of Y chromosomal genes between crossbred (*Bos taurus* × *Bos indicus*) and Indicine bulls

**DOI:** 10.1186/2049-1891-4-15

**Published:** 2013-04-04

**Authors:** Ayan Mukherjee, Gulshan Dass, Jagan Mohanarao G, Moloya Gohain, Biswajit Brahma, Tirtha Kumar Datta, Sachinandan De

**Affiliations:** 1 Animal Genomics Lab, Animal Biotechnology Centre, National Dairy Research Institute, Karnal, India; 2 Department of Veterinary Biochemistry, College of Veterinary Sciences & Animal Husbandry, Central Agricultural University, Selesih, Aizawl, Mizoram, India

**Keywords:** Absolute copy, *SRY*, *DDX3Y*, *USP9Y*, *TSPY*, Crossbred

## Abstract

**Background:**

The Y chromosome in mammal is paternally inherited and harbors genes related to male fertility and spermatogenesis. The unique intra-chromosomal recombination pattern of Y chromosome and morphological difference of this chromosome between *Bos taurus* and *Bos indicus* make it an ideal model for studying structural variation, especially in crossbred (*Bos taurus* × *Bos indicus*) bulls. Copy Number Variation (CNV) is a type of genomic structural variation that gives information complementary to SNP data. The purpose of this study was to find out copy number differences of four Y chromosomal spermatogenesis-related candidate genes in genomic DNA of crossbred and purebred Indicine bulls.

**Result:**

Four Y chromosomal candidate genes of spermatogenesis namely, *sex determining gene on Y chromosome* (*SRY*), *DEAD box polypeptide 3-Y chromosome* (*DDX3Y*), *Ubiquitin specific peptidase 9, Y-linked* (*USP9Y*), *testis-specific protein on Y chromosome* (*TSPY*) were evaluated. Absolute copy numbers of Y chromosomal genes were determined by standard curve-based quantitative real time PCR. Copy numbers of *SRY* and *TSPY* genes per unit amount of genomic DNA are higher in crossbred than Indicine bulls. However, no difference was observed in *DDX3Y* and *USP9Y* gene copy numbers between two groups.

**Conclusion:**

The present study demonstrates that the structural organization of Y chromosomes differs between crossbred and Indicine bulls which are reproductively healthy as observed from analysis of semen attributes. The absolute copy numbers of *SRY* and *TSPY* genes in unit mass of genomic DNA of crossbred bulls are significantly higher than Indicine bulls. No alteration in absolute copies of *DDX3Y* and *USP9Y* gene was found between the genome of crossbred and Indicine bulls. This study suggests that the *DDX3Y* and *USP9Y* are likely to be single copy genes in the genome of crossbred and Indicine bulls and variation in Y chromosome length between crossbred and Indicine bulls may be due to the copy number variation of *SRY* gene and *TSPY* array.

## Background

The Y chromosome plays an essential role in male sex development, spermatogenesis and male fertility [1]. The eutherian Y chromosome has unique characteristic feature that most part of this chromosome escapes meiotic recombination process with X chromosome except two regions at the tips of the X and Y chromosomes [2,3]. In absence of inter-chromosomal recombination the repeated gene sequences within a chromatid or between sister chromatids act as substrates for recombination. This unique recombination pattern of Y chromosome with its X-counterpart makes it prone to structural variation [4]. Changes in DNA content and structure are a significant source of genetic and phenotypic variation among individuals [5-8]. These types of structural variations ranging from 1 kilobase (kb) to 5 megabase (Mb) comprised mainly of copy number variation (CNV in the form of large-scale insertions and deletions), as well as inversions and translocations.

Studies on human Y chromosome has indicated the presence of 78 protein coding genes that encode 27 (18 single copy genes and 9 gene families) distinct proteins [3]. *SRY* gene encodes a protein product containing HMG box domain. The domain binds to DNA in the minor groove at a 6-base consensus target sequence and subsequently activates the cascade of testis determining pathway [9]. *DDX3Y* and *USP9Y* genes are construed as ‘fine-tuner’ of normal spermatogenesis process [10-12]. The protein product of the *TSPY* gene probably interacts with type B cyclins and activates cyclin B-CDK complexes. The activated complex, in turn, impacts on biological machineries in spermatogonial cell renewal and in prophase I spermatocyte differentiation [13].

Crossbreeding between taurine (*Bos taurus*) and indigenous cattle (*Bos indicus*) is a popular practice in Indian dairy industry. Semen of three exotic breeds namely, European Holstein Friesian, Brown Swiss and Jersey are extensively used for this purpose. The karyotypes of two bovine subspecies *Bos taurus* and *Bos indicus* have a high similarity except for the morphology of the Y chromosome [14]. Y chromosome in *Bos taurus* is submetacentric and acrocentric in *Bos indicus*. This morphological difference between *Bos taurus* and *Bos indicus* Y chromosome is the consequence of pericentric inversion [15]. In crossbred animal this difference in Y chromosome morphology may lead to small deletions or altered position between the synapse region of the X and Y chromosomes, or change in genes present on sex chromosomes. So far no study has been conducted on Y chromosomal gene copy number determination and their comparison between crossbred and Indicine bulls. Therefore, present study was designed to investigate absolute copy number differences of major Y chromosomal genes *SRY*, *DDX3Y*, *USP9Y* and *TSPY* between Karan Fries (a crossbred) and Sahiwal (purebred *Bos indicus*). We included reproductively healthy bulls in this study to avoid animals having poor semen quality which could be sequelae to the detrimental changes in Y chromosomal gene copies.

## Methods

### Animals and semen collection

All the experimental procedures involving animals were approved by the Institutional Animal Ethics Committee (IAEC), National Dairy Research Institute, Karnal, India. The seminal attributes of the bulls under study were assessed and those consistently producing good quality semen were included in the study. Semen samples were collected from 10 Karan Fries (F1 generation crossbred of Holstein-Friesian sire and Tharparkar or Sahiwal dam) and 10 Sahiwal (purebred *Bos indicus*) bulls by artificial vagina (IMV, L’Aigele cedex, France) in the early morning. The volume of the semen was measured in a graduated conical tube, mass motility was evaluated by light microscopy and sperm concentration was estimated using Haemocytometer [16] immediately after collection. The semen samples were processed for assessing the sperm quality parameters without any further delay.

### Assessment of seminal quality parameters

We assessed progressive motility, plasmalemma and acrosomal integrity of the spermatozoa to evaluate the bulls as these are the most common semen evaluation tests in cattle. The percentage of sperm showing progressive forward motility was determined by mixing 100 μL of undiluted semen into pre-warmed tubes containing 900 μL of Tris buffer (pH 7.2). A thin drop of diluted semen was placed on a pre-warmed glass slide (37°C) and evaluated under microscope. The mean of the three estimations was used as the final motility score [17]. 6-carboxyfluorescein diacetate (CFDA) and propidium iodide (PI) staining was done to assess the sperm viability as described by Selvaraju et al. [18]. Briefly, semen sample was incubated with phosphate buffered saline (PBS) based CFDA/PI staining solution at 37°C for 15 min. After incubation, 0.2% glutaraldehyde was added and stained sample were examined under magnifications of 400× using an Olympus BX51 microscope (Olympus America, Center Valley, PA, USA) equipped with CFDA filter set (excitation, 510–560 nm; emission, 505 nm). The sperm showing complete green fluorescence were considered plasmalemma intact (live), and the cells showing partial or complete red nuclei were classified as dead. The hypo osmotic swelling test was performed for assessment of sperm membrane integrity based on curled and swollen tails. The assay was performed mixing neat semen with 150 mOsm/L hypoosmotic solution (13.51 g fructose + 7.35 g trisodium citrate per liter of distilled water) and incubating at 38.5°C for 1 h [19]. After incubation, semen sample was examined under the high power magnification (400×) of a bright field microscope. Acrosomal integrity of the sperm was assessed by staining air-dried smears with fluorescein isothiocyanate-conjugated *pisum sativum* agglutinin (FITC-PSA) staining [20]. Air-dried smear was flooded with the FITC-PSA and kept in darkness for 30 min. Slides were washed in distilled water and mounted with glycerol with a cover slip. The fluorescence pattern of 200 sperm in randomly selected fields was determined under Olympus BX51 microscope (Olympus America, Center Valley, PA, USA) with 1,000× magnification. The proportions of intact, reacting, and reacted acrosome were expressed as percentages of the respective patterns in the total number of sperm counted [21].

### Collection of blood and isolation of genomic DNA

About 10 mL of blood sample was collected from each animal in sterile Vacutainer® (Beckton-Dickinson, Franklin Lakes, NJ, USA) containing sodium heparin as an anticoagulant. The collected samples were stored at 4°C and transported to the laboratory at the earliest. The genomic DNA was isolated from white blood cells using standard phenol-chloroform procedure [22]. The DNA samples were dissolved in TE buffer (pH 8.0), their concentrations were determined by optical density at 260 nm using NanoDrop 1000 Spectrophotometer (Thermo Fisher Scientific, Wilmington, DE, USA) and stored at -20°C for further use.

### Determination of absolute copy number

#### Preparation of *SRY*, *DDX3Y*, *USP9Y* and *TSPY* plasmid constructs

Fragments of *SRY* (736 bp), *DDX3Y* (2,010 bp), *USP9Y* (1,646 bp) were amplified from cDNA of peripheral blood lymphocyte. *TSPY* (1,141 bp) gene was amplified from testicular tissue of bull as the expression of the gene is testis-specific. PCR was carried out on an Eppendorf Mastercycler (Eppendorf, Hamburg, Germany) in a 25 μL reaction mixture containing 2.5 μL of 10× Paq5000 reaction buffer (provides a final Mg^+2 ^concentration of 2 mmol/L), 200 μmol/L of dNTPs (Fermentas, Lithuania), 500 nmol/L of each primer and 0.5 units of Stratagene Paq5000™ DNA polymerase (Agilent Technologies, West Cedar Creek, TX, USA). Details of the PCR primers used for amplifying these genes are listed in Table 1.

**Table 1:** Primers used to amplify the gene fragments

**Primer**	**Sequence, 5**^′^**to 3**^′^	**Melting temperature, T**_**m**_	**Product length, bp**
***SRY***	F: CGCAGTGCAGTCGTATGCTTCTGC	61°C	736
	R: GAGCGCCTTTGTTAGCGAGAGTAAGG	61°C	
***DDX3Y***	F: TTCGCGCCTTTCTTCAGGCATGAGTCA	61°C	2010
	R: CAGATTCAGTTGCCCCACCAGTCAAC	61°C	
***USP9Y***	F: TGTGGGACTCAAAAATGCTGGTGCTAC	60°C	1646
	R: ACTCCAGAAGGTATTCAGAGAAACGATTTGA	59°C	
***TSPY***	F: ATGTCGCGTCCCTTCGCCTCTGC	62°C	1141
	R: TCAGTTGTCTCTCATGGACGAACCTTCCT	62°C	

F refers to forward primer and R refers to reverse primer.

Amplified fragments were cloned separately into pcr®2.1 vector (3.9 kb) using the TOPO TA Cloning® system (Invitrogen Corporation, Carlsbad, CA, USA) and plasmids were isolated with the Qiagen Plasmid isolation kit (Qiagen GmBH, Hilden, Germany).

### Construction of standard curve

Concentrations of plasmids containing *SRY*, *DDX3Y, USP9Y* and *TSPY* inserts were adjusted at 100 ng/μL using NanoDrop 1000 Spectrophotometer (Thermo Fisher Scientific, Wilmington, DE, USA). The concentration of the plasmid was converted to corresponding copy concentration using the following equation [23]

$$\mathrm{DNA}\;\text{copy}=\frac{6.023\times 10^{23}\left(\text{copies}/\mathrm{mol}\right)\times \mathrm{DNA}\;\text{amount}\left(g\right)}{\left(\text{Plasmid}+\text{Insert}\right)\text{length}\left(\mathrm{bp}\right)\times 660\left(\mathrm{gm}/\mathrm{mol}/\mathrm{bp}\right)}$$

A tenfold dilution series of each of the plasmid constructs were used to construct the corresponding standard curves of two genes. Standard dilutions of each of the gene were assayed in triplicate. The Cp values were plotted against the logarithm of their initial template copy concentrations. Each standard curve was generated by a linear regression of the plotted points. From the slope of each curve, PCR amplification efficiency (E) was calculated according to the following equation [24]:

$$E=10^{-1/\text{slope}}-1$$

### Quantitative PCR

To determine the absolute copy number of *SRY*, *USP9Y*, *DDX3Y* and *TSPY* genes in genomic DNA samples of bulls under study the gene-specific primers were designed. The sequences of the primer sets used for real-time PCR analysis are shown in Table 2. The primers were amplified on a LightCycler® 480 instrument with software version 1.5 (Roche Diagnostics, Mannheim, Germany). Concentrations of all the DNA samples of test bulls were adjusted to 10 ng/μL. The ‘crossing point’ or Cp values were determined by ‘second-derivative max method’ in the software. All real-time PCR runs were performed in triplicate and each reaction mixture was prepared using the KAPA SYBR® FAST qPCR kit (Kapa Biosystems, Woburn, MA, USA) in a total volume of 10 μL. Reaction for *TSPY* gene comprised of 2.4 μL PCR-grade water (Sigma-Aldrich, St. Louis, MO, USA), 300 nmol/L each primer, 1× KAPA SYBR® FAST qPCR Master Mix and 2 μL of template DNA and that for *SRY*, *DDX3Y* and *USP9Y* comprised of 2 μL PCR-grade water (Sigma-Aldrich, St. Louis, MO, USA), 500 nmol/L each primer, 1X KAPA SYBR® FAST qPCR Master Mix and 2 μL of template DNA.

**Table 2:** Primers used for quantitative real time PCR

**Primer**	**Sequence, 5**^′^**to 3**^′^	**Melting temperature, T**_**m**_	**Product length, bp**
***SRY***	F: CTAGAGAATCCCAAAATGAAAAACTC	53°C	150
	R: ATATTTATAGCCCGGGTATTTGTCTC	55°C	
***DDX3Y***	F: GTTAGATTTCTGCAAATACTTGGTGTT	59°C	101
	R: GCATAGTGTCTTGTTCAATTATACGAC	60°C	
***USP9Y***	F: GTACACAGTGGTCAAGCAAGTGGTG	59°C	178
	R: CTTCTCCCATGTACTCTCCACCAAA	58°C	
***TSPY***	F: AGTTGTGAGCCCAGTTGTCA	52°C	148
	R: CACCTCCTCCACGATGTCTT	54°C	

F refers to forward primer and R refers to reverse primer.

The following cycling conditions were employed for all the genes: pre-incubation at 95°C for 3 min, followed by 40 cycles of 10 s at 95°C, 20 s at 60°C, and 1 s at 72°C. The fluorescence signal was measured at the end of each extension step at 72°C. After the amplification, a melting peak analysis with a temperature gradient of 0.1°C/s from 65 to 95°C was performed. Finally, the samples were cooled down to 40°C for 10 s.

### Statistical analysis

Results were expressed in the mean ± SEM values. Differences in seminal attributes and absolute gene copy numbers between two breeds were determined by Student’s *t*-test using Statistical Product and Service Solutions, Version 17.0.1 software (SPSS Inc., Chicago, IL, USA). A difference with *P* < 0.05 was considered statistically significant. Copy number determination experiments by real time PCR were replicated for three times.

## Results

### Seminal quality parameters

**Figure 1:**
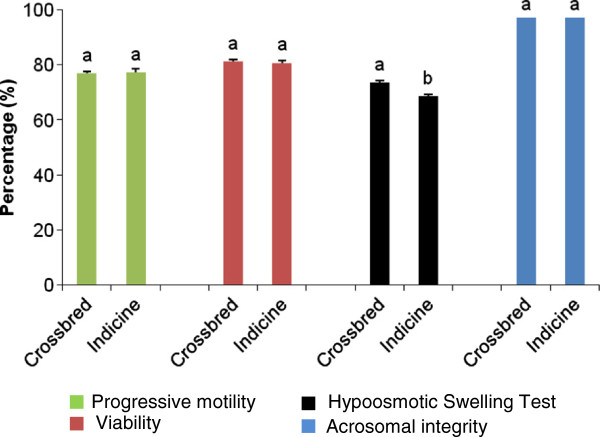
**Seminal quality parameters of crossbred and Indicine bulls.** The percentages of sperm progressive forward motility, viability, membrane integrity and acrosomal integrity were assessed in Karan Fries, a crossbred (n = 10) and Sahiwal, purebred Indicine (n = 10) bulls and the results were compared between two groups. Values are the means ± S.E.M. of three experiments performed in two groups of bulls. Different superscripts indicate significant difference (*P* < 0.05) between two groups.

The mean values of standard seminal quality parameters such as progressive forward motility, viability, membrane integrity and acrosomal integrity in the fresh semen ejaculates of Karan Fries and Sahiwal bulls have been summarized in Figure 1. No significant differences were found between semen quality parameters of Karan Fries and Sahiwal bulls like progressive motility (76.86 ± 0.79 vs. 77.2 ± 1.41), sperm viability (81.24 ± 0.92 vs. 80.83 ± 0.75), and acrosomal integrity (97.17 ± 0.26 vs. 97.16 ± 0.16). However, a significantly higher percentage of HOST-reacted spermatozoa was found in Karan Fries (73.59 ± 0.74) compared to Sahiwal (68.62 ± 0.71) bulls.

### Absolute copy number estimation of *SRY, DDX3Y, USP9Y* and *TSPY* genes

**Figure 2:**
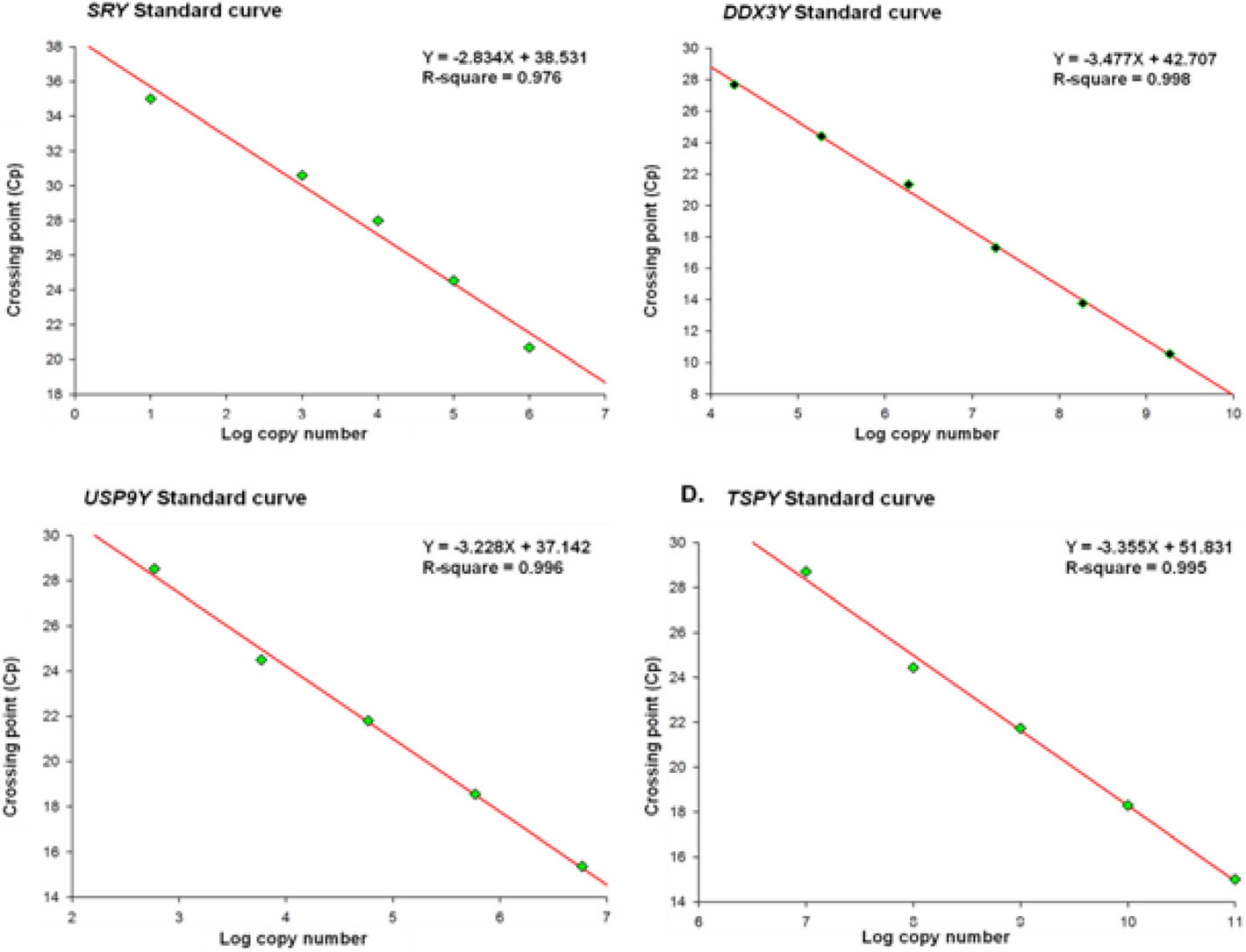
**Construction of standard curves for *SRY*, *DDX3Y*, *USP9Y *and *TSPY *quantification.** The standard curves for *SRY*, *DDX3Y*, *USP9Y* and *TSPY* absolute copy number determination together with their respective line equations are shown here. Set of serial 10-fold dilutions was made for each gene. Each of these dilutions (standard dilutions) had known copies of the plasmid construct harboring the respective gene fragment as an insert. X-axis represents log transformed values of the standard copy number and Y-axis represents the crossing point (Cp) values i.e. the fractional cycle number required for the fluorescence to cross the threshold.

As SYBR Green binds with double stranded DNA in sequence-independent manner melting curves of reaction products were analyzed to confirm that only the specific products were amplified and circumvent any issues of non-specific fluorescence. Ten-fold dilution series of recombinant plasmid DNA for all the genes ranging from 10^6 ^to 10 copies/μL for *SRY*, 1.9 × 10^9 ^to 1.9 × 10^4 ^copies/μL for *DDX3Y*, 6 × 10^6 ^to 6 × 10^2 ^copies/μL for *USP9Y* and from 10^11 ^copies/μL to 10^7 ^copies/μL for *TSPY* were prepared for generation of standard curve for quantitative analysis. The log concentration of plasmid DNA copies was plotted against the measured crossing point (Cp) values. Linear correlations between the logarithmic number of plasmid DNA copies and Cp values have been presented for all genes in Figure 2.

### Quantification of *SRY, DDX3Y, USP9Y* and *TSPY* genes in test samples

**Figure 3:**
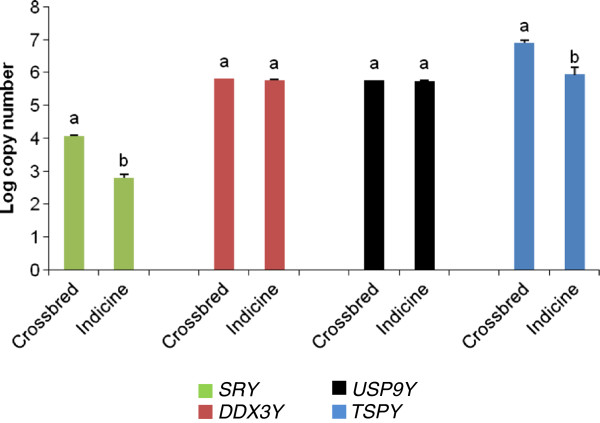
**Absolute copy numbers of *SRY*, *DDX3Y*, *USP9Y *and *TSPY *genes in genomic DNA of crossbred (n = 10) and Indicine (n = 10) bulls.** Cp values obtained for each gene from each genomic DNA sample were extrapolated in the linear equation of the respective standard curve to obtain the absolute copy number of the gene. The absolute copy was transformed in the logarithmic value. Y-axis represents the log transformed values of gene copy number. Values are the mean ± S.E.M. of three experiments performed in two groups of bulls Karan Fries (KF), a crossbred and Sahiwal, an Indicine breed. Different superscripts indicate significant difference (*P* < 0.05) between two groups.

Absolute copy concentrations in crossbred and Indicine bulls for *SRY*, *DDX3Y*, *USP9Y* and *TSPY* genes were determined from the corresponding standard curves and transformed in log_10_ values. The mean values of absolute copy concentrations of four Y- chromosomal genes in two groups of bulls have been compared in Figure 3. The mean values of *SRY* and *TSPY* in crossbred bulls (4.07, 6.9) are significantly (*P* < 0.05) higher compared to Indicine bulls (2.78, 5.93). No significant differences were observed in the log transformed absolute copy number values of *DDX3Y* and *USP9Y* gene between crossbred (5.8 and 5.75) and Indicine (5.72 and 5.74) bulls.

## Discussion

The male specific region on Y chromosome (MSY) harbors genes which have crucial role in spermatogenesis, maintenance of male germ cells and fertility. Several studies have pointed out the precise correlation between compromised sperm quality and molecular abnormalities of Y chromosome such as copy number variation of gene-rich regions. The primary objective of the current investigation was to find out the copy number differences of Y chromosomal genes between healthy crossbred and Indicine bulls. We performed semen quality parameters to select reproductively healthy bulls and to circumvent animals having poor semen quality which could be sequelae to the detrimental changes in Y chromosomal gene copies. Assessment of progressive motility, plasmalemma and acrosomal integrity are the most common semen evaluation tests in cattle [25]. Analysis of semen quality attributes shows that all the bulls under study were reproductively sound. Although percentage of HOST-reacted spermatozoa was less in Sahiwal bulls (68.62 ± 0.71) compared to KF bulls (73.59 ± 0.74) signifying better sperm membrane integrity in KF bulls but it does not affect the overall fertility potential of Sahiwal bulls as these bulls are regularly used for breeding program. Present study was conducted to find out whether Y chromosomal genes *SRY*, *DDX3Y*, *USP9Y* and *TSPY* copies vary in genomic DNA of crossbred and Indicine bulls. We found significantly lower number of *SRY* and *TSPY* gene copies in Indicine bulls compared with crossbred bulls. Absolute copies of *DDX3Y* and *USP9Y* gene did not vary between these two breeds*.* The bulls included in the present study were not paternally related. Hence, the copy numbers in different genes on Y chromosomes may be identical by state but not by descent. To our knowledge this is the first report of absolute quantitative PCR to measure levels of Y chromosomal gene copy numbers in genomic DNA samples of bovine. Compared to relative PCR methods, the use of absolute real-time PCR allows a direct comparison of the exact copy number of the genes in each genomic DNA sample [26]. The absolute quantitative PCR reported here estimates the copy numbers of the gene of interest without the requirement for a reference gene through the construction of standard curve.

Absolute copy number of *SRY* is significantly higher in crossbred bulls compared to Indicine bulls. *SRY* is a single copy gene in the genome of most mammalian species. But multiple copies of this gene have also been reported in cat, rat and rabbit and it is not clear whether multiple copies of this gene are present on cattle Y chromosome [27]. Copy number of *TSPY* gene varies substantially even between closely related mammals like human and chimpanzees [28]. In the present study log *TSPY* copy number varied widely from 5.48 to 6.85 in Indicine and from 6.31 to 7.13 in crossbred reflecting wide variation of this multi-copied gene within the bovine species. Studies conducted so far in human have pointed out the association of *SRY, DDX3Y*, *USP9Y* and *TSPY* genes with spermatogenesis and seminal quality [29-33]. However, the impact of copy number variation of these genes on reproductive parameters is still elusive. This finding is congruent with the previous report in human [31]. The phenotypic differences among different phyla or classes of organisms result from accumulation of mutations in the genome and their subsequent stabilization within the genome for the sake of adaptation to different environments [34]. So it may be assumed that normal functionality requirement of the genes in two different subspecies are different. Accordingly the genes attained that optimum level in *Bos taurus* and *Bos indicus* genome. As *DDX3Y* is a single copy gene in the cattle genome [27] the absolute copy number of *DDX3Y* gene should not vary between two bovine subspecies. Present study also did not reveal any significant variation between crossbred and Indicine bulls in terms of absolute copy number of *DDX3Y* gene. Although *USP9Y* is a single copy gene in most of the mammalian species the exact copy number of the gene in the cattle genome is not known [27]. No significant difference in absolute copy number of this gene between crossbreed and Indicine bulls was observed and quantity of this gene is almost equal to *DDX3Y* gene copy number. So, it can be assumed that like other mammalian species cattle Y chromosome also harbors single copy of this gene.

Y chromosome is highly heterogeneous in both size and genetic makeup among species [28,35]. Vast structural polymorphisms have been detected in both heterochromatin and euchromatin of Y chromosome. Significant variations have been reported in the length of Y chromosome in different breeds of cattle [36] and *Bos taurus* × *Bos indicus* cattle [37]. The Y chromosome length variation observed in the present study may be due to variation in copies of *TSPY* array. A previous study of localization of *TSPY* on *Bos taurus* Y chromosome showed precise position of *TSPY* array on Yp arm [38]. Acrocentric *Bos indicus* Y has distinctly smaller p-arms than submetacentric *Bos taurus* Y [39]. The same study [39] proposed the phenomena of pericentric inversion with breakpoints on the proximal p-arms and on the distal band q12 of *Bos indicus* Y. It may be speculated that loss of *TSPY* gene copies occurred during the point of divergence of Y chromosome in two subspecies.

## Conclusion

This study suggests that the structural organization of Y chromosome varies between crossbred and Indicine bulls which are reproductively healthy as observed from assessment of semen attributes. Absolute copy number of *DDX3Y* and *USP9Y* do not vary between these two bovine subspecies probably because of their single-copy presence in the genome. *TSPY*, a multicopy gene in bovine genome, varies substantially between crossbred and Indicine bulls. Variation in Y chromosome length between crossbred and Indicine bulls might be due to the copy number variation of *TSPY* array and *SRY* gene. These findings establish a foundation for association study between chromosomal genes copy number and reproductive fitness in crossbred and Indicine bulls and existence of any correlation between them will be valuable in determining breeding programs.

## Competing interests

None of the authors have any competing interest to declare.

## Authors’ contributions

All listed authors have made substantial contributions to: the research design, analysis or interpretation of data and in drafting the paper. All authors read and approved the final manuscript.
